# Oropharyngeal Gonorrhea in Absence of Urogenital Gonorrhea in Sexual Network of Male and Female Participants, Australia, 2018

**DOI:** 10.3201/eid2507.181561

**Published:** 2019-07

**Authors:** Vincent J. Cornelisse, Catriona S. Bradshaw, Eric P.F. Chow, Deborah A. Williamson, Christopher K. Fairley

**Affiliations:** Melbourne Sexual Health Centre, Carlton, Victoria, Australia (V.J. Cornelisse, C.S. Bradshaw, E.P.F. Chow, C.K. Fairley);; Monash University, Melbourne, Victoria, Australia (V.J. Cornelisse, C.S. Bradshaw, E.P.F. Chow, C.K. Fairley);; The University of Melbourne, Parkville, Victoria, Australia (D.A. Williamson)

**Keywords:** gonorrhea, *Neisseria gonorrhoeae*, disease transmission, transmission, infectious, pharynx, Australia, sex network, oropharyngeal gonorrhea, urogenital gonorrhea, STIs, sexually transmitted infections, kissing, bacteria, whole-genome sequencing

## Abstract

We describe a sexual network consisting of 1 nonbinary-gendered participant and 2 male and 4 female participants in Australia, 2018. Six of 7 participants had oropharyngeal gonorrhea in the absence of urogenital gonorrhea. This observation supports a new paradigm of gonorrhea transmission in which oropharyngeal gonorrhea can be transmitted through tongue kissing.

Oropharyngeal gonorrhea is considered to be acquired primarily from an infected penis during oral sex (*1*). However, male urethral gonorrhea is usually symptomatic (*2*–*4*), prompting men to seek treatment soon after symptoms appear (*5*), resulting in short duration of infectivity and low point prevalence. Thus, infected penises are unlikely to be the source to explain the observed high prevalence of oropharyngeal gonorrhea (*6*,*7*). To address this epidemiologic conundrum, we previously described a paradigm of gonorrhea transmission in which oropharyngeal gonorrhea can be acquired from a partner’s oropharynx during tongue kissing (*8*), as originally proposed in the 1970s and 1980s (*9*,*10*). However, investigating whether kissing can lead to gonorrhea transmission has been difficult because kissing often occurs concurrently with other sexual acts (*11*). We describe a sexual network of 1 nonbinary, 2 male, and 4 female participants who were tested for gonorrhea at genital and oropharyngeal sites in early 2018 to explore gonorrhea transmission dynamics.

## The Study

Ethics approval was obtained from the Alfred Hospital Ethics Committee, Melbourne, Australia (project no. 462/18). The index case was identified during routine patient care at Melbourne Sexual Health Centre (Carlton, Victoria, Australia). After patients consented to take part in our study, they contacted their sexual partners, who then each consented and were interviewed. Participants independently provided accounts of their sexual activity to permit interparticipant verification. We describe the timing of events with respect to day 0, the day of a music festival during which most sexual activity occurred.

We tested for *Neisseria gonorrhoeae* infection by nucleic acid amplification with the Aptima Combo 2 assay and confirmed by the Aptima GC assay (Gen-Probe, https://www.hologic.com). We performed whole-genome sequencing and bioinformatic analyses on available samples (Appendix).

Recalled accounts of sexual activity were consistent between participants. No participant reported symptoms of gonorrhea, and none used antimicrobial drugs during the relevant period.

On day 10, the index patient (participant 1 [P1], nonbinary gender, assigned female sex at birth) sought screening for sexually transmitted infections at Melbourne Sexual Health Centre. Though asymptomatic, P1 tested positive for oropharyngeal gonorrhea and negative for urogenital gonorrhea. P1’s most recent negative test for gonorrhea was 5 months prior. Between the previous negative test and day 0, P1 had sex with 4 men besides their primary male sexual partner (P2) (Figure). These 4 other male sexual partners subsequently tested negative for gonorrhea; however, we were not able to confirm what anatomic sites were tested. On day 0, P1 had tongue kissed P3 (female) and had tongue kissed and had reciprocal orogenital sex (without condoms) and penovaginal sex (without condoms) with P2.

**Figure:**
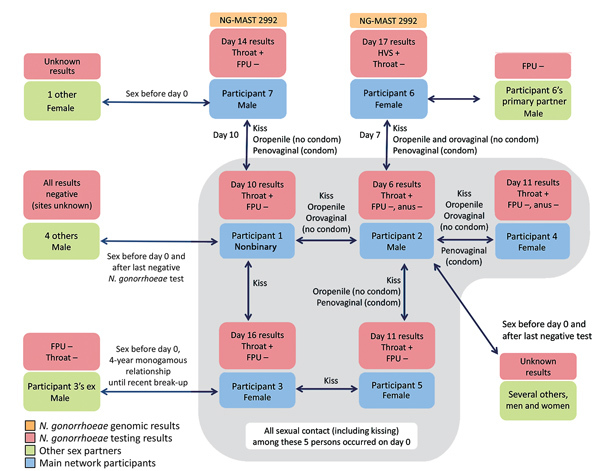
*Neisseria gonorrhoeae* diagnoses among participants of a sexual network, Australia, 2018. FPU, first-pass urine; HVS, high vaginal swab; NG-MAST, *N. gonorrhoeae* multiantigen sequence type.

On day 16, P3 tested positive for oropharyngeal gonorrhea and negative for urogenital gonorrhea. She had not been tested for gonorrhea in the past 4 years because, until a recent break-up, she had been in a long-term monogamous relationship. She had no other sexual contacts (including kissing) with other men or women during this time. P3’s expartner was later contacted and tested and was negative for oropharyngeal and urogenital gonorrhea.

On day 6, before P1 underwent testing, P2 sought routine asymptomatic screening for sexually transmitted infections at Melbourne Sexual Health Centre and tested positive for oropharyngeal gonorrhea and negative for urogenital and anal gonorrhea. P2’s most recent test was 4 months earlier, when he tested negative for oropharyngeal, anal, and urogenital gonorrhea. P2 had sex with several men and women besides his primary partner between his last test and day 0; test results are not known for many of these sex partners.

On day 0, P2 had sex with P4 (female), consisting of tongue kissing, reciprocal orogenital sex without condoms, and penovaginal sex with condoms. On day 11, P4 tested positive for oropharyngeal gonorrhea and negative for urogenital and anal gonorrhea. Eleven days before day 0, P4 had tested negative for oropharyngeal and urogenital gonorrhea. P2 and P4 had had sex weekly for 5 months before day 0.

On day 0, P2 also had sex with P5 (female), consisting of tongue kissing, oropenile sex without condoms, and penovaginal sex with condoms. On day 11, P5 tested positive for oropharyngeal and negative for urogenital gonorrhea. P5’s previous gonorrhea test (negative results) was 1–2 years earlier. P5 also tongue kissed P3 but had no other sexual contact with her. P5 had no other sexual contacts, including tongue kissing, the 3 months before day 0.

On day 7, P2 had sex with P6 (female), consisting of tongue kissing, reciprocal orogenital sex without condoms, and penovaginal sex with condoms. On day 17, P6 tested positive for urogenital gonorrhea but negative for oropharyngeal gonorrhea. P6 had tested negative for urogenital gonorrhea ≈3 weeks before her contact with P2. The only other person (male) P6 had sex with during the time between her negative and positive test results subsequently tested negative for urogenital gonorrhea.

On day 10, P1 had sex with P7 (male), consisting of penovaginal sex with condoms, tongue kissing, and oropenile sex without condoms. P1 and P7 had sex weekly for 2 months before day 0. On day 14, P7 tested positive for oropharyngeal and negative for urogenital gonorrhea. His previous test was 4 years prior. P7 had 1 other sexual partner (female) in the months before day 0, and she was unable to be contacted.

Two *N. gonorrhoeae* isolates (from P6 and P7) were available for whole-genome sequencing. Both were *N. gonorrhoeae* multiantigen sequence type 2992, and no single-nucleotide polymorphism differences were found between the isolates (BioProject no. PRJNA449254).

This report describes a sexual network consisting of 1 nonbinary participant and 2 male and 4 female participants, of which 6 participants had oropharyngeal gonorrhea in the absence of urogenital gonorrhea. Although it is possible that some of the oropharyngeal infections were caused by undisclosed sexual contacts or inaccurate testing information, an additional explanation is that gonorrhea was transmitted by tongue kissing.

Two gonorrhea samples were available for genomic analysis and were highly related genomically. These participants were separated in this network by 2 other participants, corroborating the epidemiologic observation that these infections were the result of within-network transmission rather than a result of sexual contact with persons external to the network. Also, given the low prevalence of gonorrhea among the general population in Melbourne (https://kirby.unsw.edu.au/sites/default/files/kirby/report/SERP_Annual-Surveillance-Report-2017_compressed.pdf), the probability that all participants acquired gonorrhea from external partners is low.

No men in this network had urethral gonorrhea, suggesting that the oropharynx-to-penis route has a lower transmission probability than tongue kissing. This finding is consistent with an existing mathematical model that included transmission by kissing, which calculated a per-act transmission of 1% for oral sex and 17% for kissing (*12*). Few observational studies have examined transmission by kissing, but 1 study of male couples found 26% concordance of oropharyngeal gonorrhea between partners (*13*).

Because this report describes sexual contacts that occurred at a music festival, participants’ recall might have been affected by alcohol or drugs. Also, awareness of being part of a study involving sexual partners could have affected participants’ willingness to disclose information. However, recall was consistent between participants, suggesting that their recall was accurate.

## Conclusions

Accumulating evidence suggests that tongue kissing might be a common mode of gonorrhea transmission (*12*–*14*). The observation that expectorated saliva from persons with oropharyngeal gonorrhea contains high loads of *N. gonorrhoeae* DNA suggests a plausible mechanism for transmission (*15*). The sexual network described here adds to this evidence. We also highlight the need for routine screening for oropharyngeal gonorrhea for all persons with multiple sexual partners.

Appendix Supplementary methods to investigate oropharyngeal gonorrhea in the absence of urogenital gonorrhea in a sexual network of male and female participants, Australia, 2018.
