# Supplementary material for: Oropharyngeal Gonorrhea in Absence of Urogenital Gonorrhea in Sexual Network of Male and Female Participants, Australia, 2018
Source: Emerg Infect Dis. 2019 Jul;25(7):1373–6. doi: 10.3201/eid2507.181561 (PMC6590767; doi:10.3201/eid2507.181561)
Supplement: Appendix — Supplementary methods to investigate oropharyngeal gonorrhea in the absence of urogenital gonorrhea in a sexual network of male and female participants, Australia, 2018. [file 18-1561-Techapp-s1.pdf]

Article DOI: https://doi.org/10.3201/eid2507.181561

Oropharyngeal Gonorrhea in Absence of Urogenital Gonorrhea in Sexual Network of Male and Female Participants, Australia, 2018

Appendix

Supplementary Methods

DNA Extraction and Whole-Genome Sequencing

DNA extraction and whole-genome sequencing were performed at the Microbiological Diagnostic Unit Public Health Laboratory at the University of Melbourne (Carlton, Victoria, Australia). Genomic DNA was isolated from a single colony by using the QIAsymphony SP platform (QIAGEN, https://www.qiagen.com). Whole-genome sequencing was performed using the Illumina (https://www.illumina.com) NextSeq platform with 150-bp paired-end reads. All sequencing reads are available on the National Center for Biotechnology's Sequence Read Archive under BioProject no. PRJNA520805.

Bioinformatic Analysis

We analyzed sequences with Snippy version 4 (Seeman T, https://github.com/tseemann/snippy). In brief, reads were trimmed to remove adaptor sequences and low-quality bases with Trimmomatic (*1*), and Kraken (v0.10.5-β) was used to investigate for contamination (*2*). Reads were aligned to the NCCP11945 reference genome (*3*) with snippy v4. De novo assemblies were also performed with SPAdes (v.3.9.0) (*4*), as part of the Nullarbor pipeline, with genes annotated by using Prokka (v.1.12-β, Seemann T, https://github.com/tseemann/prokka). *Neisseria gonorrhoeae* multiantigen sequence type results were inferred in silico by using NGMASTER (*5*).

References

1. Bolger AM, Lohse M, Usadel B. Trimmomatic: a flexible trimmer for Illumina sequence data. Bioinformatics. 2014;30:2114–20. PubMed http://dx.doi.org/10.1093/bioinformatics/btu170

2. Wood DE, Salzberg SL. Kraken: ultrafast metagenomic sequence classification using exact alignments. Genome Biol. 2014;15:R46. PubMed http://dx.doi.org/10.1186/gb-2014-15-3-r46

3. Chung GT, Yoo JS, Oh HB, Lee YS, Cha SH, Kim SJ, et al. Complete genome sequence of *Neisseria gonorrhoeae* NCCP11945. J Bacteriol. 2008;190:6035–6. PubMed http://dx.doi.org/10.1128/JB.00566-08

4. Bankevich A, Nurk S, Antipov D, Gurevich AA, Dvorkin M, Kulikov AS, et al. SPAdes: a new genome assembly algorithm and its applications to single-cell sequencing. J Comput Biol. 2012;19:455–77. PubMed http://dx.doi.org/10.1089/cmb.2012.0021

5. Kwong JC, Gonçalves da Silva A, Dyet K, Williamson DA, Stinear TP, Howden BP, et al. *NGMASTER*: in silico multi-antigen sequence typing for *Neisseria gonorrhoeae*. Microb Genom. 2016;2:e000076. PubMed http://dx.doi.org/10.1099/mgen.0.000076
